# Primary immunodeficiencies (PID) Life Index in Southeast Asia: A comparative analysis of PID Principles of Care (PoC)

**DOI:** 10.3389/fimmu.2023.1151335

**Published:** 2023-03-30

**Authors:** Chee Mun Chan, Nizar Mahlaoui, Silvia Sánchez−Ramón, Martine Pergent, Leire Solis, Johan Prevot, Adli Ali

**Affiliations:** ^1^ Department of Pediatric, Faculty of Medicine, Universiti Kebangsaan Malaysia, Kuala Lumpur, Malaysia; ^2^ Research Center, Hospital Tunku Ampuan Besar Tuanku Aishah Rohani, Universiti Kebangsaan Malaysia (UKM) Specialist Children’s Hospital, Kuala Lumpur, Malaysia; ^3^ Pediatric Immunology-Hematology and Rheumatology Unit, Necker Children’s University Hospital, Assistance Publique-Hôpitaux de Paris (AP-HP), Paris, France; ^4^ French National Reference Center for Primary Immune Deficiencies (CEREDIH), Necker Children’s University Hospital, Assistance Publique-Hôpitaux de Paris (AP-HP), Paris, France; ^5^ Department of Clinical Immunology, Instituto de Medicina del Laboratorio (IML) and Instituto de Investigación Clínico San Carlos (IdISSC), Hospital Clínico San Carlos, Madrid, Spain; ^6^ Department of Immunology, ENT and Ophthalmology, Complutense University School of Medicine, Madrid, Spain; ^7^ The International Patient Organization for Primary Immunodeficiencies, Brussels, Belgium; ^8^ Institute of IR4.0, Universiti Kebangsaan Malaysia, Bangi, Malaysia; ^9^ Infection and Immunology Health and Advanced Medicine Cluster, Universiti Kebangsaan Malaysia, Cheras, Kuala Lumpur, Malaysia

**Keywords:** primary immunodeficiencies, Southeast Asia, Life Index, Principles of Care, universal health care

## Abstract

**Objective:**

To analyze the implementation of the Principles of Care (PoC) in primary immunodeficiencies (PID) in Southeast Asia (SEA) countries - six years after its call of action.

**Methodology:**

Using the newly developed PID Life Index software, the index of implementation of principles of care in the management of PIDs patients involving the six participating SEA countries (Cambodia, Indonesia, Malaysia, Vietnam, Thailand, and Philippines) were extracted. For each of the six separate principles, the index from the six countries will be compared and presented based on the calculated index.

**Results:**

Comparative analysis of the six principles of care of PID in the SEA countries showed low diagnostic rate with minimal availability of diagnostic tests options. Generally, almost all SEA countries provide curative treatments, vaccines, and anti-infectious therapies although the reimbursement scheme varied in relieving patients’ financial burden. We also highlighted the active involvement of patient organizations in SEA, with main areas of work focused on advocacy and increasing awareness among public and healthcare professionals.

**Discussion and conclusion:**

It is applaudable that the SEA continent is gradually strengthening its work in management of PID, especially in Thailand and Vietnam. However, more emphasis must be placed among stakeholders in SEA countries towards successful implementation of the PoC for a holistic management of PID patients.

## 1 Introduction

Primary immunodeficiencies (PID) are a heterogeneous group of inherited immune system defects, encompassing over 450 different disorders (1). Despite their label as rare disorders, PID affect an estimated six million individuals worldwide, with 70 to 90% of cases remain undiagnosed (2). Some PID disorders are present more commonly than others and together, they significantly impact the lives of those affected, with an overall global prevalence rate of approximately 1 in 10, 000 individuals (1, 2). With impaired immune system rendering susceptibility to germs such as bacteria, viruses, fungi, protozoa, and tumor cells, PID patients tend to be at higher risk for autoimmune diseases, infections and dysregulated inflammation (1, 3). Depending on the parts of the affected immune system, PID are classified into different groups based on the underlying pathology, which allows for specific treatments to be initiated. This is a crucial aspect of PID management, since treating only the symptoms without addressing the underlying cause can result in further deterioration of a patient’s health (3). Over 50% of individuals with PID present with antibody deficiencies, necessitating treatment to replace absent antibodies. However, more severe PID patients with cellular defects of lymphocytes may require hematopoietic stem cell transplantation (3). Timely and accurate diagnosis is crucial, as delayed or missed diagnosis of PID often result in high morbidity and mortality rates. Recent studies suggest that the prevalence of PID may be higher than previously thought due to missed diagnoses (2). Thus, raising knowledge and awareness among healthcare professionals and the public is crucial for early disease detection and optimal treatment, ultimately leading to improved patient outcomes.

The International Patient Organization for Primary Immunodeficiencies (IPOPI), has developed a global gold standard framework for PID care, aimed at ensuring early diagnosis and access to appropriate treatments for all patients worldwide, through collaboration among a multidisciplinary team of specialists from different countries. Led by Helen Chapel et al. in 2014, they established six principles of care for PID patients including the role of specialized centers, the importance of registries, the need for multinational research, the role of patient organizations and the importance of sustained access to all treatments (3). Subsequently in 2020, PID Life Index was introduced by an IPOPI expert task force to measure the implementation of PID Principles of Care worldwide (4). To date, the revised six principles in the PID Life Index are: i) Availability of Diagnosis; ii) National Patient Organization; iii) National Registries; iv) National Specialized centers; v) Availability of Treatment; vi) Universal Health Coverage. Each principle was described and structured through a series of measurable criteria. Although there were different criteria for each principle, they were formulated according to their respective domains, resulting in the final 6 principles having relatively equal weightage. This allows readers to observe progress or changes in the implementation of each of the principle independently, with a higher score indicating an improved PID environment. The PID Life Index, a user-friendly web-based tool, visualizes these principles and allows users to navigate and prioritize them according to their needs. This web-based tool is the first to offer a comprehensive and holistic overview of the global PID environment. It is hoped to guide stakeholders and policy makers in ensuring that PID patients are diagnosed and adequately treated, thereby enabling them to lead productive lives worldwide (4).

Asia Pacific is the largest continent, comprising of 48 countries with approximately 4.6 billion inhabitants, accounting for 60% of the global population (5). However, compared to the western world, several countries in Asia are still underdeveloped, resulting in wide discrepancy in healthcare facilities, standard of living and education. Specifically, in Southeast Asia (SEA) countries, a systematic review by Rajah et al. reported a mean prevalence of 55.3% of limited health literacy (6). Health literacy, commonly defined as an individuals’ ability to read, understand, and apply health information to make informed decisions about their healthcare, is highly associated with mortality rates and non-adherence to medication, contributing to a higher economic burden on healthcare (6). This creates a socio-economic barrier that impedes understanding of their health, leading to failure in self-management of their disease and poorer outcomes. Interestingly, the review noted that one in every two participants in SEA had limited health literacy, which was much higher than a United States-based study whereby one in every four participants were found to have such issues (6). Besides, there is also a distinct ethnic diversity among these 11 countries of SEA, which sets them apart from East Asian countries like China, Japan and Korea which are more ethnically homogenous (7–9). Uniquely in SEA countries, the disease phenotype of PID is rather unusual as its initial presentations are not commonly described in classical literature (10). Studies have reported higher incidence of *Mycobacterium tuberculosis*, *Mycobacterium bovis*, *Chromobacteriumviolaceum, Burkholderiapseudomallei* and *Penicillium marneffei*, which are endemic in SEA countries (10, 11). Therefore, in cases where live vaccines such as Bacillus Calmette-Guerin (BCG) are administered to severely immunodeficient infants in SEA countries, they may experience complications, often leading to death with a high mortality rate of 43.5% (10, 12). Consequently, a family history of early infant death may serve as a warning sign for family members to seek prompt medical attention and prevent similar PID-related complications in subsequent children (13). With that, we concur that increasing awareness and knowledge are crucial to ensure proper implementation of principles of care, given that political and financial factors play a significant role in improving the situation (14). To date, limited studies have examined the implementation of principles of care for PID patients in SEA, mainly due to many regional challenges. Among commonly cited were insufficient training and lack of expertise, diagnostic technical and financial difficulties, inadequate facilities for treatments and the lack of patient organizations. Therefore, our objective is to create a regional baseline data by outlining the principles of care, which are currently lacking in the region, and emphasizing their importance for future studies. Thus, this study is the first to describe and discuss the landscape of the PID environment in different SEA countries, providing a much-needed overview of the general status of the principles of care in the region.

## 2 Materials and method

Our study analyzed data extracted from the PID Life Index on a virtual platform. We explored the index from 6 SEA countries namely i) Cambodia ii) Indonesia iii) Malaysia iv) Thailand v) Philippines and vi) Vietnam. Descriptive analysis was applied throughout this study and the results were expressed in n (%), where n represented the total number of countries involved in each principle. The score of each principle was assessed separately and tabulated into tables and figures for regional comparison. This study presents the findings of data collected in 2022 from six SEA countries, providing a comprehensive overview of general status of the Principles of Care in the region.

## 3 Results

### 3.1 Principle 1: Diagnosis of PID in the world

**Figure 1:**
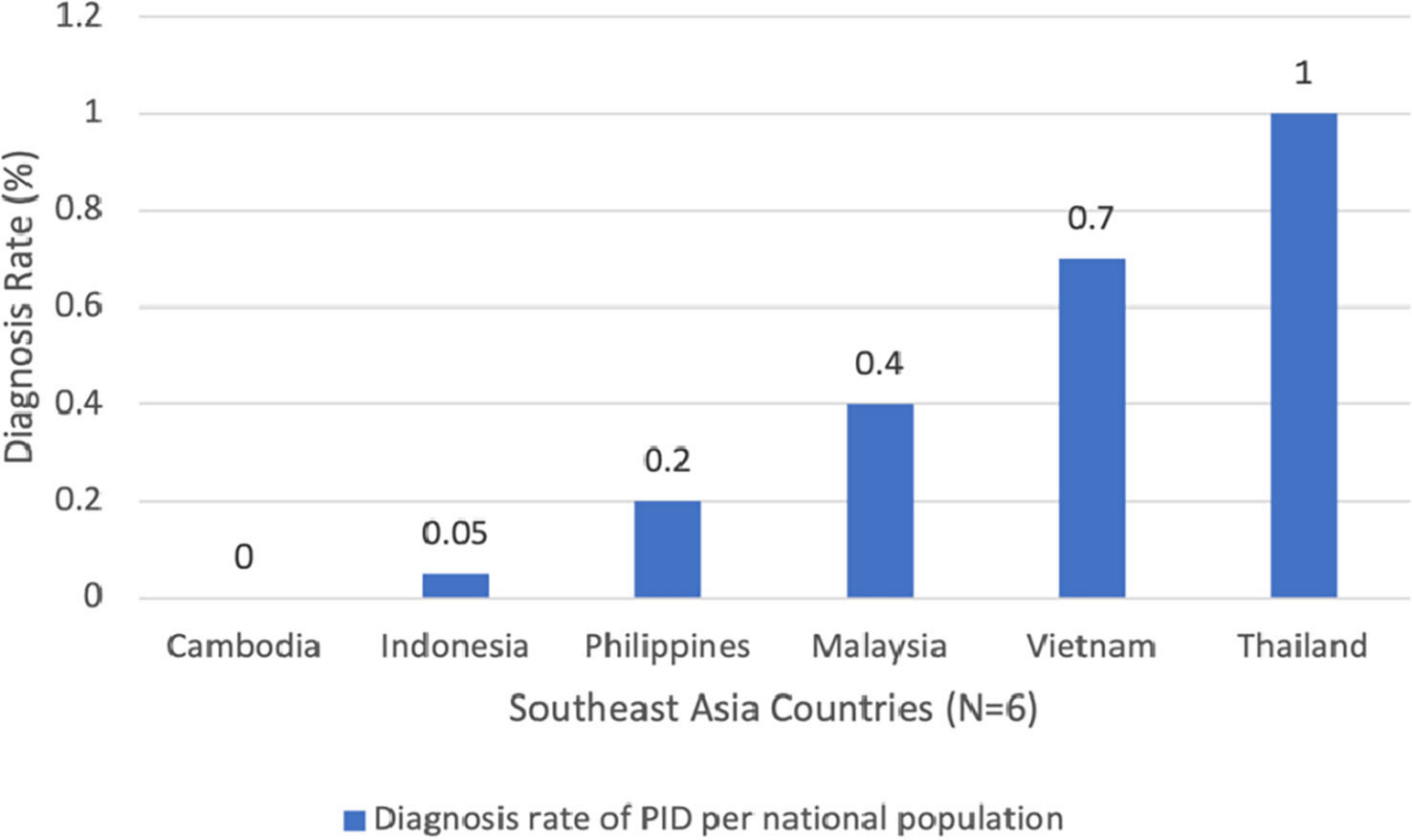
Diagnosis rate of Primary immunodeficiencies (PID) in Southeast Asia (SEA). Notes: The diagnosis rate of different countries are calculated based on the known number of patients and the theoretical number of PID patients in each country (based on a prevalence rate of 1 in 2, 000 inhabitants). The diagnosis rate is expressed in %.

The first principle of PID diagnosis includes the availability of i) biological tests, ii) genetic diagnosis, iii) prenatal tests, and iv) newborn screening. A country is considered to be able to provide the best standard of diagnosis when it has access to all four different modalities (1). In addition, the PID Life Index measures the diagnosis rate of different countries, calculated based on the known number of patients and the theoretical number of PID patients in each country (based on a prevalence rate of 1 in 2, 000 inhabitants) (5). According to the index, five out of six SEA countries, except Cambodia, provide at least one type of diagnostic test to their citizens. Generally, the diagnosis rate of PID is very low compared to other Asian countries, with Thailand recording the highest rate at 1.1%, while Cambodia had 0% diagnosis rate, as depicted in **Figure 1**. Among the four options, the most frequently used test is biological diagnosis. Malaysia is the only SEA country that has full availability of biological diagnosis, while Cambodia does not have any availability of the four diagnostic tools analyzed.

#### 3.1.1 Severe combined immunodeficiencies (SCID) newborn screening

Although there is growing evidence supporting the implementation of newborn screening for early detection of severe combined immunodeficiencies (SCID), most countries in SEA have not yet adopted this screening program. Over the years, active detection was mainly done in families who had already lost a previous child due to SCID. Within the six SEA countries, only Vietnam has regional implementation of the SCID newborn screening. The only other SEA country which had started on SCID newborn screening program is Singapore, however it is not included in our analysis as Singapore is not part of the contributing countries for PID Life Index. Nonetheless, through the Singapore’s experience, within a span of one year, the newborn screening program (NBS) had successfully identified 13 cases of non-SCID T-cell lymphopenia using T-cell receptor excision circle (TREC) assay performed in dried blood spots of 35888 newborns (15).

### 3.2 Principle 2: Availability of treatments for PID patients worldwide

As PID can be categorized depending on the nature of defect, every PID patient requires different types of treatments. There are two groups of treatments, namely supportive (e.g. immunoglobulin replacement therapy, anti-infectious therapies) and curative (bone marrow transplant, gene therapy or thymic transplant). In the PID Life Index, a therapy is considered to be available if they have received a market authorization or can be readily accessed by PID patients in the country.

#### 3.2.1 Biological treatments

The PID Life Index assesses 7 types of biological treatments. These treatments include: (i) thrombopoietin receptor agonists; (ii) C1 inhibitor concentrate; (iii) growth factors (G-CSF, GM-CSF, EPO); (iv) cytokines/interleukins (Interleukin alpha, interferon gamma, IL-2, IL-7, etc); (v) monoclonal antibodies; (vi) immunosuppressants and immunomodulators; and (vii) enzyme replacement therapy for ADA SCID. All six SEA countries have their own practice of treatment modalities, hence there is not much of commonality observed between them. Malaysia and Vietnam have the greatest number of biological treatments availability, which included growth factors, immunosuppressors/immunomodulators and monoclonal antibodies. Among these three options, growth factors were commonly given and also available in the Philippines as the only accessible biological treatment. Otherwise, the other three countries cited irregular to no practice of such treatment options. On the other hand, enzyme replacement therapy for ADA SCID, C1-inhibitor concentrate and thrombopoietin receptor agonist were the least used treatments, with no availability in all countries except Vietnam, of which the only country reported the use of the latter.

#### 3.2.2 Immunoglobulins

Antibody deficiencies are common in more than 50% of the PID patients (16). The mainstay of treatment for these patients is replacement of the absent or low antibodies through immunoglobulin replacement therapy (IRT). Patients are given IRT at regular intervals to increase serum IgG trough levels to physiologic concentrations for protection against infections (17). To date, three types of IRT administration are commercially available which are intravenous (IVIg), subcutaneous (SCIg) and facilitated subcutaneous infusion (fSCIg). It is important for patients to consider the different routes of IRT to ensure lifelong adherence to treatment as they transition through different phases of life. The WHO Essential Medicines List also labeled immunoglobulin as essential therapies in the treatment for PID in both adult and pediatric populations (18). Fortunately, in SEA, IRT was widely practiced in all countries except Cambodia. The preferred route for all 5 countries was *via* intravenous administration. Additionally, Malaysia, Thailand, and Vietnam also offered IRT by subcutaneous route (SCIg), although they were not regularly used (**Table 1**). One of the published applications of SCIg was the case report of a 12-year-old Malaysian patient with X-linked agammaglobulinemia (XLA) who demonstrated the successful resolution of bronchiectasis after conversion from IVIg to SCIg (19). Hypothetically, SCIg has advantages over IVIg as they present with lesser associated systemic effects like anaphylactic reaction and less pharmacokinetic fluctuation to ensure a more stable serum IgG trough levels throughout the interval period. Therefore, the adoption of subcutaneous route in SEA countries is noteworthy, particularly when patients encounter challenges with venous access.

**Table 1:** Types of treatments available for Primary Immunodeficiencies (PID) patients in Southeast Asia (SEA).

Types of treatment	Availability of treatment, N=6 (%)			
	No	Not regularly	Most of the time	Yes
A) Biological treatments				
Enzymes	0	0	0	6 (100.0)
Immunosuppressors	1 (16.7)	3 (50.0)	1 (16.7)	1 (16.7)
Monoclonal antibodies	0	4 (66.7)	1 (16.7)	1 (16.7)
Cytokines	3 (50.0)	3 (50.0)	0	0
Growth Factor	1 (16.7)	2 (33.3)	1 (16.7)	2 (33.3)
C1 inhibitor	5 (83.3)	1 (16.7)	0	0
Thrombopoietin	4 (66.7)	1 (16.7)	0	1 (16.7)
B) Routes of immunoglobulin				
IVIG	0	1 (16.7)	-^a^	5 (83.3)
SCIG	3 (50.0)	2 (33.3)	-^a^	1 (16.7)
FSCIG	4 (66.7)	2 (33.3)	-^a^	0
C) Anti-infectious				
Antibiotics	0	0	1 (16.7)	5 (83.3)
Antivirals	0	0	0	6 (100.0)
Antifungals	0	2 (33.3)	1 (16.7)	3 (50.0)
Antiparasitic	1 (16.7)	1 (16.7)	0	4 (66.7)
D) Vaccines	1 (16.7)	-^a^	-^a^	5 (83.3)
E) Curative treatments				
Bone marrow transplant	2 (33.3)	-^a^	-^a^	4 (66.7)
Gene therapy	6 (100.0)	-^a^	-^a^	0
Thymic transplant	6 (100.0)	-^a^	-^a^	0

a Not determined.

Descriptive analysis was used to compare the frequency of different PID treatment options in 6 SEA countries. The results were expressed in n (%), where n represented the number of countries.

#### 3.2.3 Anti-infectious and vaccine prophylaxis

Infections commonly manifest among PID patients, thus anti-infectious therapies are widely used. These include a broad range of treatments, such as antibiotics, antivirals, antifungals and antiparasitic. They are usually given as prophylaxis to prevent serious infections or therapeutically to treat active infections. **Table 1** demonstrates that all SEA countries provided anti-infectious prophylaxis and is readily available. The most common prophylaxis were antibiotics and antivirals, which were given to almost all PID patients. Additionally, Malaysia, Thailand, and Vietnam also provided antifungal and antiparasitic prophylaxis, while Philippines and Indonesia did not practice as such.

Similarly, vaccines also protect against serious and potentially life-threatening infectious diseases. However, greater caution must be exercised when administering vaccines to PID patients, as live-attenuated vaccines may revert to virulence in these individuals. The WHO recognizes immunization as a fundamental element of primary health and an undeniable human right. Consequently, vaccination is also widely available for PID patients in all countries except Indonesia, as shown in **Table 1**.

#### 3.2.4 Curative treatments

Ultimately, the curative interventions available for PID patients are hematopoietic stem cell transplantation (HSCT), gene therapy or thymic transplant. Since PID are rare and often unique in each patient, defining a universal transplant regime is difficult (20). In the SEA region, HSCT is currently the only curative treatment available and is practiced in four countries, with Cambodia and Indonesia being the exceptions. As of now, gene therapy and thymic transplant are not available in SEA due to their high costs of treatment and the requirement for highly specialized teams and infrastructures (3, 21).

### 3.3 Principle 3: Universal health coverage

Despite the availability of a wide array of available treatments and diagnostic tests, access to them is not universal for all PID patients, and financial challenges can be a barrier as shown **Table 2**. As depicted by the PID Life Index, affordability of PID care for patients and families remains an obstacle, especially in low to middle-income countries. As a result, financially constrained PID communities may be unable to undergo diagnostic tests or receive adequate therapies, refraining them from achieving the highest possible standard of PID care.

**Table 2:** Universal health coverage of different therapies for patients with Primary Immunodeficiencies (PID).

Types of coverage	Reimbursement level, N=6 (%)					
	Not applicable^a^	Paid fully by patient or patient’s family	Up to 50%	From 51 to 69%	From 70 to 90%	From 91 to 100%
A) Biological treatment						
Enzymes	5 (83.3)	1 (16.7)	0	0	0	0
Immunosuppressors	2 (33.3)	2 (33.3)	1 (16.7)	0	1 (16.7)	0
Monoclonal antibodies	1 (16.7)	3 (50.0)	2 (33.3)	0	0	0
Cytokines	2(33.3)	3 (50.0)	1 (16.7)	0	0	0
Growth Factor	1 (16.7)	1 (16.7)	1 (16.7)	0	2 (33.3)	1 (16.7)
C1 inhibitor	5 (83.3)	1 (16.7)	0	0	0	0
Thrombopoietin	3 (50.0)	2 (33.3)	0	0	1 (16.7)	0
B) Routes of immunoglobulin						
IVIG	-^b^	2 (33.3)	0	0	4 (66.7)	0
SCIG	-^b^	3 (50.0)	1 (16.7)	0	1 (16.7)	0
FSCIG	4 (66.7)	0	2 (33.3)	0	0	0
C) Anti-infectious						
Antibiotics	-^b^	2 (33.3)	0	0	3 (50.0)	1 (16.7)
Antivirals	-^b^	2 (33.3)	0	0	3 (50.0)	1 (16.7)
Antifungals	-^b^	2 (33.3)	0	0	3 (50.0)	1 (16.7)
Antiparasitic	-^b^	2 (33.3)	0	0	2 (33.3)	2 (33.3)
D) Vaccines	1 (16.7)	2 (33.3)	0	1 (16.7)	1 (16.7)	1 (16.7)
E) Curative treatments						
Bone marrow transplant	2 (33.3)	1 (16.7)	1 (16.7)	1 (16.7)	1 (16.7)	0
F) Diagnosis testing						
Biological	2 (33.3)	2 (33.3)	2 (33.3)	0	2 (33.3)	0
Genetic	2 (33.3)	3 (50.0)	2 (33.3)	0	0	0
Prenatal	3 (50.0)	2 (33.3)	2 (33.3)	0	0	0

a Indicates that the treatment options were not available in the country, hence had no data on the coverage level.

b not determined.

Descriptive analysis was used to compare the coverage level of different PID treatment options in 6 SEA countries. We stratified the coverage level into five different groups and the results were expressed in n (%), where n represented the number of countries.

#### 3.3.1 Coverage of reimbursement of biological treatments

Firstly, this study examines the countries of Malaysia and Vietnam, which reported frequent practice of biological treatments. Malaysia is laudable for recording the highest reimbursement cost (91 to 100%) of growth factors to patients, with Vietnam following closely behind (70 to 90%). However, the other costs of biological modalities like immunosuppressors and monoclonal antibodies, were fully paid by patients or caregivers in Malaysia. In contrast, Vietnam still reimbursed from 50 to 70% of these costs. Patients from the remaining SEA similarly bear the full cost of their biological treatments.

#### 3.3.2 Coverage of reimbursement for immunoglobulin (Ig) therapies for patients with PID

More than 50% of PID patients received Ig therapies, which are not only costly but also require lifelong administration for most patients in comparison to other treatment modalities. Thus, sustaining the coverage level is crucial to maintain a good level of PID care (22). It is essential to recognize the barriers that prevent continuous access to therapies and increase advocacy efforts among various organizations to emphasize the importance of Ig therapy to their stakeholders (1). As reported earlier, all countries except Cambodia practice the use of IRT. Being the most frequent route of Ig administration globally, it is commendable that four countries in the region reimbursed 70 to 90% of the cost to all patients on IVIg, setting them apart from Philippines where the cost was fully paid by the patients themselves. Additionally, Thailand covered up to 50% of the cost for SCIg treatment, further demonstrating the effort to improve access to Ig therapies.

#### 3.3.3 Coverage of reimbursement for anti-infectious therapies and vaccine prophylaxis for patients with PID

All countries, except for Indonesia, provide some level of reimbursement for anti-infectious prophylaxis programs, with Malaysia, Thailand, and Vietnam covering 70 to 90% of the cost for all four types of anti-infectious regimes. Vaccination reimbursement was highest in Malaysia, covering 91 to 100% of the cost, while Vietnam and Thailand provided 70 to 90% and 51 to 69% coverage, respectively. However, both Philippines and Cambodia did not offer any subsidy of prophylaxis therapies to their patients.

#### 3.3.4 Coverage of curative therapies for patients with PID

According to the PID Life Index, over half of the SEA countries have expertise for HSCT as the only curative treatment (4). Of the four countries, Vietnam had the highest reimbursement rate at approximately 70 to 90%, followed by Malaysia (51 to 69%) and Thailand (up to 50%). It is important to note that HSCT is a costly procedure as it involves not only the medical procedure but also logistic expenses such as traveling and hospitalization fees, pharmaceutical costs, diagnostic costs, and also indirect expenses such as lost income due to absenteeism from work to care for PID patients (23). Unfortunately, despite these financial challenges, Philippines did not have any reimbursement scheme for curative treatment.

#### 3.3.5 Coverage of reimbursement for diagnostic tests for PID

The coverage of diagnostic tests differs greatly across the world, depending on the type of test (1). Thailand recorded the highest diagnosis rate, possibly attributed by the highest rate of reimbursement especially for biological testing (70 to 90%) and up to 50% for the other two modalities. This is also similar in Vietnam, although they only reimbursed for biological testing (70 to 90%). A similar privilege is not extended to other countries in the SEA region, as families in those have to cover the cost of the tests themselves.

#### 3.3.6 Overall universal health coverage in SEA

**Figure 2:**
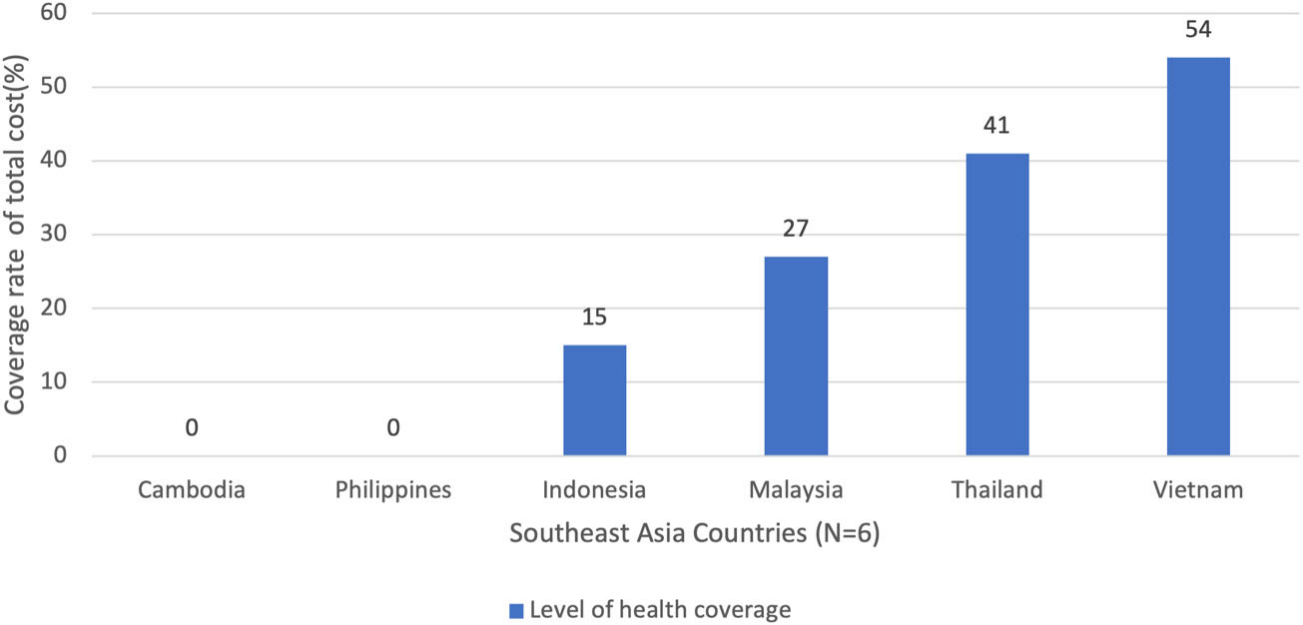
Overall universal health coverage in six Southeast Asia (SEA) countries. Notes: This data was derived from the summary of PID Life Index which demonstrated the coverage level of treatment from its total cost in each country. The coverage rate was expressed in percentage (%).

In conclusion, our analysis indicates that Vietnam and Thailand have the highest health coverage rates for PID patients, with 54% and 41%, respectively. This is consistent with the reimbursement efforts seen in most of the categories. Malaysia and Indonesia, on the other, have lower coverage rates of 27% and 15% respectively, which is half of the coverage level compared to Vietnam and Thailand. Therefore, these countries should expand their coverage scheme and increase reimbursement rates for the care of PID patients. Nonetheless, we find it commendable that four out of six countries have active health coverage, as shown in **Figure 2**, with the exception of Philippines and Cambodia, for which no available data were reported for this principle.

### 3.4 Principle 4: Specialized centers

The availability of specialized centers is important in ensuring that patients with PID have access to medical and nursing expertise regardless of their location (1). Although these centers will vary depending on the available resources and expertise, nevertheless, there should be internationally agreed standards of care which offer a holistic approach to diagnosis, treatment, and care of patients with PID. According to the established criteria in the principles of care, pediatric and adult centers (including transition care) should have specialized diagnostic and management services, and able to provide training and professional development including facilities to conduct related research (3, 4). On top of that, specialists should also actively engage patients with other hospital specialties to ensure integrated care for better treatment outcomes. Hence, an integrated network is necessary to connect these centers nationwide with a formalized referral pathway for diagnosis, treatment and follow-up.

It is noteworthy that only two SEA countries, namely Philippines and Thailand, have several centers for both pediatric and adult PID services, as well as national PID specialized network (**Table 3**). While Indonesia has established several national PID specialized centers, only one center across the country offers adult PID service. In contrast, Vietnam offers adult PID service at several centers but has only one national network of specialized centers. However, they are still ahead of Malaysia which does not provide any adult PID service and only has several uncoordinated small PID specialized networks.

**Table 3:** Availability of specialized centers for Primary Immunodeficiencies (PID) patients.

Types of specialized centers	Availability of facilities, N=6 (%)					
	No	Yes, only one center	Yes, several uncoordinated small networks	Yes, single national network of specialized centers	Yes, some specialized centers	Yes, several centers
Adult PID service	2 (33.3)	1 (16.7)	0	0	0	3 (50.0)
National PID specialized centre/network	1 (16.7)	0	1 (16.7)	1 (16.7)	0	3 (50.0)
Transition care	0	0	0	0	3 (50.0)	0

Descriptive analysis was used to compare the availability of different types of specialized centers in six SEA countries. We stratified them into six different groups of facility availability. The results were expressed in n (%), where n represented the number of countries.

Besides that, transition care facilities are only available in some specialized centers in Indonesia, Vietnam, and Thailand. This may be attributed to the shortage of adult care services and adult immunologists who are equipped to provide care to pediatric patients transitioning into adulthood. Therefore, additional efforts should be made to ensure that adult PID patients receive the same level of care as pediatric patients. We agree that appropriate management for adult patients is pivotal not only to prevent unnecessary future suffering and complications, but also to ensure comprehensive and cost-effective long-term care.

### 3.5 Principle 5: National patient organizations

Over the years, national patient organizations for PID have become an increasingly important stakeholder in the decision-making process for the care of rare diseases. It is generally acknowledged that patient representatives are well-informed of their conditions and treatment, thus able to provide personal perspectives which are unique in advocating for their community. Aside from creating a supportive environment for patients and family members to exchange experiences and receive guidance, many organizations also actively advocate to improve diagnosis, treatment, and care for patients in individual countries (24). The PID principle of care cited that “all countries should aim to have an efficient national patient organization, representing all PID patients – children and adults – in order to give them a voice and represent their interest in policy making” (3).

**Figure 3:**
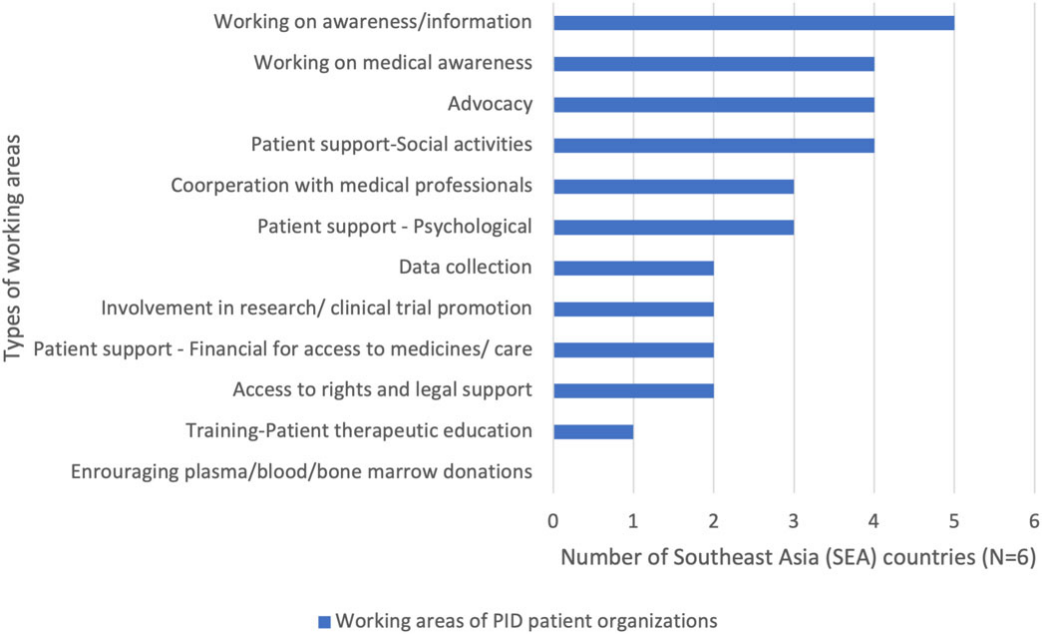
Main working areas for Primary Immunodeficiencies (PID) patient organization in Southeast Asia (SEA). Notes: The PID Life Index identified 12 working areas by patient organizations. We used descriptive analysis to compare the frequency of types of working areas in SEA. The results were expressed in n (%), where n represented the number of countries.

All SEA countries except Cambodia indicated that they have established a national patient support group. Among the 5 countries, the national patient organization in Vietnam demonstrated to have the largest working areas of activities (90%), followed by the Philippines (78%). We further stratified the working areas of the five countries as depicted in **Figure 3**. Sharing the same goal, these organizations were actively engaged in raising awareness about PID. They also imparted information to the medical and public community to reduce diagnostic delay, increase diagnostic rates and improve knowledge of the diseases. Besides that, almost all of the organizations emphasized the importance of providing patient support, which included organizing social activities and offering psychological support. With the exception of Thailand, four countries were also engaged in advocacy work such as the introduction of newborn screening for SCID. Unfortunately, only Indonesia and Vietnam were actively collecting data for research or clinical trials in the field of research. It is also worth highlighting that only Vietnam provided training for patients to educate them about therapeutic aspects and options in the country. Nevertheless, it is important to note that at the time of this report, none of the SEA countries had any paid professional staff to assist in their work.

### 3.6 Principle 6: National registries

Given the limited available data for epidemiological studies, the establishment of national registries is important to evaluate the proportion of affected individuals among the general population (prevalence), as well as record the number of new cases diagnosed each year (incidence) (3). Additionally, these registries play a crucial role in identifying regional areas with low diagnostic rates, providing insights on possible diagnostic delays associated with increased morbidity and mortality (25). National and regional registries are therefore essential in any public health program, providing necessary data for planning services, public health monitoring, research, and care of patients. Despite their importance, many countries globally, including those in SEA, lack registries (38 countries based on the PID Life Index) (1). Regionally, only Malaysia and Vietnam have bone marrow registries, while none have a national PID registry, which is consistent with most other countries.

## 4 Discussion

Generally, in the field of primary immunodeficiencies and rare diseases, patient organizations, medical experts, and stakeholders often face the lack of comprehensive data, which hampers the understanding of the situation in a given country (5). Despite the revolution of information technology in many aspects of our daily lives, its application to the health sector is still scarce (26). Hence, the development of PID Life Index constitutes the first virtual technology initiative that displays data on a set of comprehensive principles which describes the level of care for PID patients in different countries (5). Unfortunately, there are wide discrepancies between accessibility and affordability throughout the world despite the global scientific advancement with an increasing number of diagnostic tools and better treatment options (1). To date, although some principles of care for PID are being actively implemented in many countries worldwide, data from the PID Life Index data shows that no single country surveyed had yet attained a 100% score based on the index. Nonetheless, many of these principles are interlinked, and improvements in one area could lead to improvements in others. Therefore, it is commendable that SEA countries are making gradual efforts to achieve this goal.

### 4.1 Medical education and supporting network

Regionally in SEA, we noted an urgent need for medical education. Many countries cited lack of immunologists, putting many PID patients at risk of being undiagnosed or misdiagnosed which resulted in serious health implications and mortality. Studies have demonstrated that a lack of knowledge among healthcare professionals and inadequate training for immunologists has contributed to the low diagnosis rate of PID across the world (27). The United Nations Development Program 2019 reported that Cambodia was inadequately equipped with expertise and resources for diagnosis and management of PID (28). Healthcare professionals from Cambodia also expressed difficulties in identifying and categorizing PID, hence possibly explained the country’s poor performance in all of PID principles of care (28). There were no diagnostic tools and treatment available in this country except supportive therapies (anti-infectious and vaccine prophylaxis) which were fully funded by patients. Similarly in Malaysia, Intan et al. found that only 51 PID cases were detected in the span of 20 years, supporting the low diagnosis rate of 0.4 which was very low compared to other Asian countries (29). Our findings from the PID Life Index agreed to the conceptualized idea that this could be attributed to the unavailability of PID patient database registry, inadequate immunodiagnostic capacity, unavailability of national newborn screening program and reduced awareness among healthcare workers on early detection of PID. As of 2022, there were only seven clinical immunologists in Malaysia, of which six were centered in the heart of Kuala Lumpur. This clearly depicts the geographical barriers to accessing standard healthcare, compounded by the insufficient number of trained clinical immunologists for Malaysia’s population of 1 to 6.48 million. Clinical immunology is still not recognized as a subspecialty field in Malaysia, further aggravating the situation and posing more challenges to improving the care of PID patients (30).

### 4.2 Poor access to diagnostic tests and treatments

From the PID Life Index, poor access to diagnosis and treatment was a major concern for many national PID patient organizations, particularly for adult patients (1). The lack of infrastructure to perform more advanced diagnostic tests such as genetic testing and treatments like HSCT, were cited as fundamental problems by the patient organizations (5). HSCT, as previously mentioned, was not widely available in many parts of SEA. From 1983 to 2018, only 20 PID patients underwent HSCT in Malaysia. Delay in diagnosis was reported as one of the major hindrances toward the success of HSCT, which otherwise could contribute up to a 90% survival outcome (30). Interestingly, things had started to change positively over the last 5 years, with Vietnam documented the highest increase in number of HSCT performed in Asia Pacific in the past decade, showing a promising future in the curative option for PID patients in the region (31).

### 4.3 Affordability and sustainability

Although most of the SEA PID patients have access to immunoglobulins and anti-infectious therapies, not all countries cover the cost for their patients. On a positive note, Vietnamese healthcare administration have standardized the reimbursement rate of approximately 70 to 90% for all treatment and diagnosis costs to relieve the burden of PID patients. This is similarly observed in Thailand, whereby the highest country’s reimbursement (70 to 90%) was allocated to biological testing, anti-infectious prophylaxis and IVIg treatment. Despite the small number of diagnosed PID patients (Diagnostic rate = 0.05) in Indonesia, it is applaudable that the healthcare system is concerned about the welfare of their patients by subsidizing 70 to 90% of the treatment cost and heavily reimbursing anti-infectious prophylaxis (91 to 10%). Contradictory, Philippines is the only SEA country that has no reimbursement scheme for any of the treatments offered to their patients. This is supported by Carol et al., who highlighted that only 4 out of 6 B-cell deficient patients had the financial means to undergo genetic studies (32). This financial barrier is further illustrated when all of these patients were unable to comply with the recommended interval of IVIg every three to four weeks, hence reducing the efficacy of the treatment (32).

Additionally, the sustainability of Ig treatment has recently emerged as a problem in many countries worldwide. A study by Toh et al. reported that a large amount of IVIg usage was for off-label indications (47%), which may potentially contribute to shortage of IVIg supplies for primary indicated diseases like PID (33). This is further supported by Lee and colleagues, who demonstrated approximately MYR 2.3 million was spent on off-label use of IVIg in two tertiary hospitals [57.5%], indicating a huge allocation of budget for its non-licensed indications (34). Therefore, a recommendation for a strict approval system and policies to monitor the dispensing of IVIg is required to ensure optimal usage of this valuable resource worldwide.

### 4.4 The role of regional network in specialized training and advocacy

Ideally, international and regional collaboration amongst healthcare professional is fundamental in supporting training and medical education, while widening the access to the latest and cutting-edge technology in improving diagnosis, treatments, and research in the field of PID (1). The existing PID organizations and networks in this region are the Southeast Asia Primary Immunodeficiency (SEAPID) network established in 2014 and the Asia Pacific Society for Immunodeficiencies (APSID), conceptualized in Osaka in 2015 (5). Annually, APSID collaborates with international faculties to arrange educational events that involve postgraduate students from various Asian countries to impart knowledge about the present clinical and practical challenges encountered in the region. Additionally, Malaysia, Indonesia, Thailand, Philippines, and Vietnam have each established national networks to address the issues of providing training, education, and advocacy in the field of PID.

Furthermore, in 2010, a global awareness campaign, the World Primary Immunodeficiencies Week (WPIW) was launched to instill awareness on PID (4). Since then, it has become an annual event that has successfully achieved its goals of raising awareness and improving diagnostic rates (35). Additionally, these organizations provide regular updates to the PID community about the latest developments in this field, including medical advances, political regulatory decisions, supply, and safety of the treatments (4). As such, it is imperative to prioritize patient-centricity and involve patients in developing personalized treatment plans to ensure a gold standard of long-term care for individuals with PID.

We identified several limitations in our study. Firstly, the utilization of PID Life Index, which is a crucial tool in gathering the data on the landscape of PID in SEA, may have some shortcomings. Although data was gathered systematically through IPOPI’s network of national member organizations or medical contact in countries where no patient organization existed, some respondents may have considered this as an opportunity to promote their country’s progress in the field of PID. This bias is also evident when the Index does not generalize the overall situation in the country, especially in regions where only one medical expert was known. Thus, this shortcoming was not able to provide an overall perspective in a given country. Secondly, our findings relied solely on data from the PID Life Index, without expert validation in the involved SEA countries. Given the scarcity of available studies on the epidemiological status of PID in SEA, it was difficult to fully grasp the implementation of these principles. Thirdly, we did not enquire further about the national policies in-place for PID patients, nor did we explore the existing discrepancies between national laws and the day-to-day reality of PID patients. Therefore, we were unable to justify the current implementation of health policies in a given country for PID patients and their families. Lastly, our study did not encompass the entire SEA region, as national data from countries such as Singapore, Myanmar, Laos, and Brunei were not included in the PID Life Index database.

## 5 Conclusion

The study has highlighted several challenges, particularly the large undiagnosed patient population, poor access to appropriate treatment, and financial sustainability which adversely affect the quality of life for PID patients in the SEA region. However, the availability of different diagnostic tools and treatment options are becoming more accessible and affordable for PID patients in many SEA countries, attributed to the increased advocacy roles of patient organizations and healthcare professional networks, as well as awareness about primary immunodeficiencies among the public and healthcare professionals. Nevertheless, we acknowledge that the PID stakeholders in SEA should collaborate and catalyze various parties to ensure full implementation of the principles of care within the shortest possible period. We recognize the PID Life Index as an important tool in providing an integrated worldwide view of the actual situation of PID principles of care. Nonetheless, the data from the Index not only provides a benchmark for information sharing, but also serves as a basis for awareness and advocacy purposes. Therefore, this virtual tool is ideal support for analyzing the current global PID environment and its future prospect.

## Data availability statement

The original contributions presented in the study are included in the article/supplementary material. Further inquiries can be directed to the corresponding author.

## Author contributions

AA initiated the conceptual framework, provided expert advice for the study design, and supervised the study. CMC was responsible for literature review, study design development, data extraction and analysis, and drafted the manuscript. AA, NM, SS-R, MP, LS, and JP reviewed and edited the manuscript. All authors contributed to the article and approved the submitted version.
